# Inflammatory IFIT3 renders chemotherapy resistance by regulating post-translational modification of VDAC2 in pancreatic cancer

**DOI:** 10.7150/thno.43093

**Published:** 2020-06-01

**Authors:** Zhefang Wang, Jie Qin, Jiangang Zhao, Jiahui Li, Dai Li, Marie Popp, Felix Popp, Hakan Alakus, Bo Kong, Qiongzhu Dong, Peter J. Nelson, Yue Zhao, Christiane J. Bruns

**Affiliations:** 1 Department of General, Visceral, Tumor and Transplantation Surgery, University Hospital Cologne, Kerpener Straße 62, 50937 Cologne, Germany.; 2 Department of General, Visceral und Vascular Surgery, Ludwig-Maximilian-University (LMU), 81377 Munich, Germany.; 3 Department of Anesthesiology, Changhai Hospital, Naval Medical University, Shanghai, PR China.; 4 Department of Surgery, Klinikum Rechts der Isar, Technische Universität München, Munich, Germany.; 5 Department of General Surgery, Huashan Hospital & Cancer Metastasis Institute & Institutes of Biomedical Sciences, Fudan University, Shanghai, China.; 6 Medizinische Klinik und Poliklinik IV, University of Munich, Munich, Germany.

**Keywords:** PDAC, Chemotherapy resistance, IFIT3, VDAC2, post-translational modification

## Abstract

Pancreatic ductal adenocarcinoma (PDAC) is one of the most lethal cancers worldwide and effective therapy remains a challenge. IFIT3 is an interferon-stimulated gene with antiviral and pro-inflammatory functions. Our previous work has shown that high expression of IFIT3 is correlated with poor survival in PDAC patients who receive chemotherapy suggesting a link between IFIT3 and chemotherapy resistance in PDAC. However, the exact role and molecular mechanism of IFIT3 in chemotherapy resistance in PDAC has been unclear.

**Methods:** A group of transcriptome datasets were downloaded and analyzed for the characterization of IFIT3 in PDAC. Highly metastatic PDAC cell line L3.6pl and patient-derived primary cell TBO368 were used and IFIT3 knockdown and the corresponding knockin cells were established for *in vitro* studies. Chemotherapy-induced apoptosis, ROS production, confocal immunofluorescence, subcellular fractionation, chromatin-immunoprecipitation, co-immunoprecipitation and mass spectrometry analysis were determined to further explore the biological role of IFIT3 in chemotherapy resistance of PDAC.

**Results:** Based on PDAC transcriptome data, we show that IFIT3 expression is associated with the squamous molecular subtype of PDAC and an increase in inflammatory response and apoptosis pathways. We further identify a crucial role for IFIT3 in the regulation of mitochondria-associated apoptosis during chemotherapy. Knockdown of IFIT3 attenuates the chemotherapy resistance of PDAC cells to gemcitabine, paclitaxel, and FOLFIRINOX regimen treatments, independent of individual chemotherapy regimens. While IFIT3 overexpression was found to promote drug resistance. Co-immunoprecipitation identified a direct interaction between IFIT3 and the mitochondrial channel protein VDAC2, an important regulator of mitochondria-associated apoptosis. It was subsequently found that IFIT3 regulates the post-translational modification-O-GlcNAcylation of VDAC2 by stabilizing the interaction of VDAC2 with O-GlcNAc transferase. Increased O-GlcNAcylation of VDAC2 protected PDAC cells from chemotherapy induced apoptosis.

**Conclusions:** These results effectively demonstrate a central mechanism by which IFIT3 expression can affect chemotherapy resistance in PDAC. Targeting IFIT3/VDAC2 may represent a novel strategy to sensitize aggressive forms of pancreatic cancer to conventional chemotherapy regimens.

## Introduction

Pancreatic ductal adenocarcinoma (PDAC) is the fourth leading cause of cancer death in Europe, with a 5-year overall survival of less than 7% 1,2. Most patients are diagnosed at a late stage with locally advanced or metastatic diseases that are ineligible for surgical resection - at present the only potentially curative option 3. Of the patients that receive resection, most will eventually face recurrence and metastasis 4. To date, chemotherapy represents the only additional option to control pancreatic cancer and its benefits are limited due in large part to drug resistance emerging during treatment 2. Recently, the combination of nab-paclitaxel with gemcitabine 5 and the four drugs regimen FOLFIRINOX 6 was shown to improve the overall survival of PDAC patients as compared to gemcitabine alone. Despite this enhanced survival found through combination chemotherapy, PDAC remains highly resistant to the various drugs currently available and thus remains a major clinical challenge.

Chemotherapy resistance in PDAC has been shown to be driven by various mechanisms, including intrinsic aberrant gene expression (e.g. NF-κB pathway, IAPs and Bcl-2) and extrinsic tumor microenvironment effectors (e.g. cancer-associated fibroblasts and extracellular proteins) 2,7. NF-κB is a mediator of inflammation-associated cancers and is frequently observed to be upregulated in PDAC 8. The persistent activation of NF-κB is associated with chemotherapy resistance in PDAC 8,9.

The recent application of integrated genomic analysis has helped to identify molecular subtypes of PDAC. The two major PDAC subtypes characterized to date are represented by the “classical” form and the “basal-like” form, each showing differing responses to chemotherapy drugs 10,11. The basal-like subtype, also referred to as a squamous or quasi-mesenchymal subtype, is associated with a poor prognosis in PDAC patients 11. These tumors show enrichment in activated gene networks associated with inflammation, the hypoxia response, and metabolic reprogramming. These findings emphasize the importance of inflammatory pathways in progression and chemotherapy resistance of PDAC.

Interferon-induced protein with tetratricopeptide repeats 3 (IFIT3) is an inflammatory-associated gene regulated through the JAK/STAT signaling pathway that shows aberrant expression in PDAC 12. The IFIT protein family is comprised of RNA-binding proteins that promote the inhibition of translation of non-self RNA during viral infection 13. The presence of multiple tetratricopeptide repeat (TPR) motifs promotes the homodimerization of IFIT3 or its heterodimerization with other IFIT proteins 13. In addition, an interaction of IFIT3 with the transcription factor STAT1 14, the mitochondrial protein MAVS 15, and the innate immune response protein STING 16 have also been reported. While most reports have focused on the antiviral role of IFITs, emerging evidence has suggested that these proteins may also play a role in cancer progression 14,17. Yang et al. suggested that IFIT3 expression may predict the interferon-α response in hepatocellular carcinoma patients 14. Pidugu et al. reported that IFIT1 and IFIT3 could promote oral squamous cell carcinoma (OSCC) metastasis as well as contribute to cisplatin and 5-FU chemotherapy resistance 18,19. IFIT1 and IFIT3 were shown to enhance p-EGFR recycling leading to increased sensitivity to gefitinib in OSCC cells 17. Previously, our group has reported that IFIT3 can be up-regulated in PDAC and that high expression of IFIT3 can correlate with poor survival in PDAC patients who received chemotherapy strongly suggesting an association of IFIT3 with chemotherapy resistance in PDAC 20. To date, the molecular basis of IFIT3 in PDAC chemotherapy resistance is unknown. It has been reported that IFIT3 may act as a bridge between MAVS and TBK1 during the antiviral response suggesting a role for IFIT3 in regulation of mitochondria-associated apoptosis 15.

Voltage-dependent anion-selective channel protein 2 (VDAC2) is a channel protein found in the mitochondrial outer membrane that controls the transport of metabolites across the mitochondrial outer membrane and also plays a crucial role in regulating mitochondria-associated apoptosis 21. While an interaction between VDAC2 and BAK/BAX protein is widely accepted, the mechanism of VDAC2 as a modulator of mitochondria-associated apoptosis remains controversial 22. Cheng et al. 23 reported that VDAC2 can interact with an inactive conformation of BAK and through this inhibit mitochondria-associated apoptosis. Chin et al. 24 reported that VDAC2 is crucial for BAX-mediated apoptosis. However, how VDAC2 is regulated in the context of PDAC has not been addressed.

The present study focused on the potential molecular mechanisms by which IFIT3 may foster chemotherapy resistance in PDAC cells. Importantly, for the first time, we describe the interaction of IFIT3 with VDAC2. IFIT3 was found to modulate the post-translational modification (PTM) level of VDAC2 by effecting O-GlcNAcylation, and to thus, protect PDAC cells from chemotherapy induced apoptosis independent of the chemotherapy regimen.

## Results

### Expression and characterization of IFIT3 in PDAC

**Figure 1:**
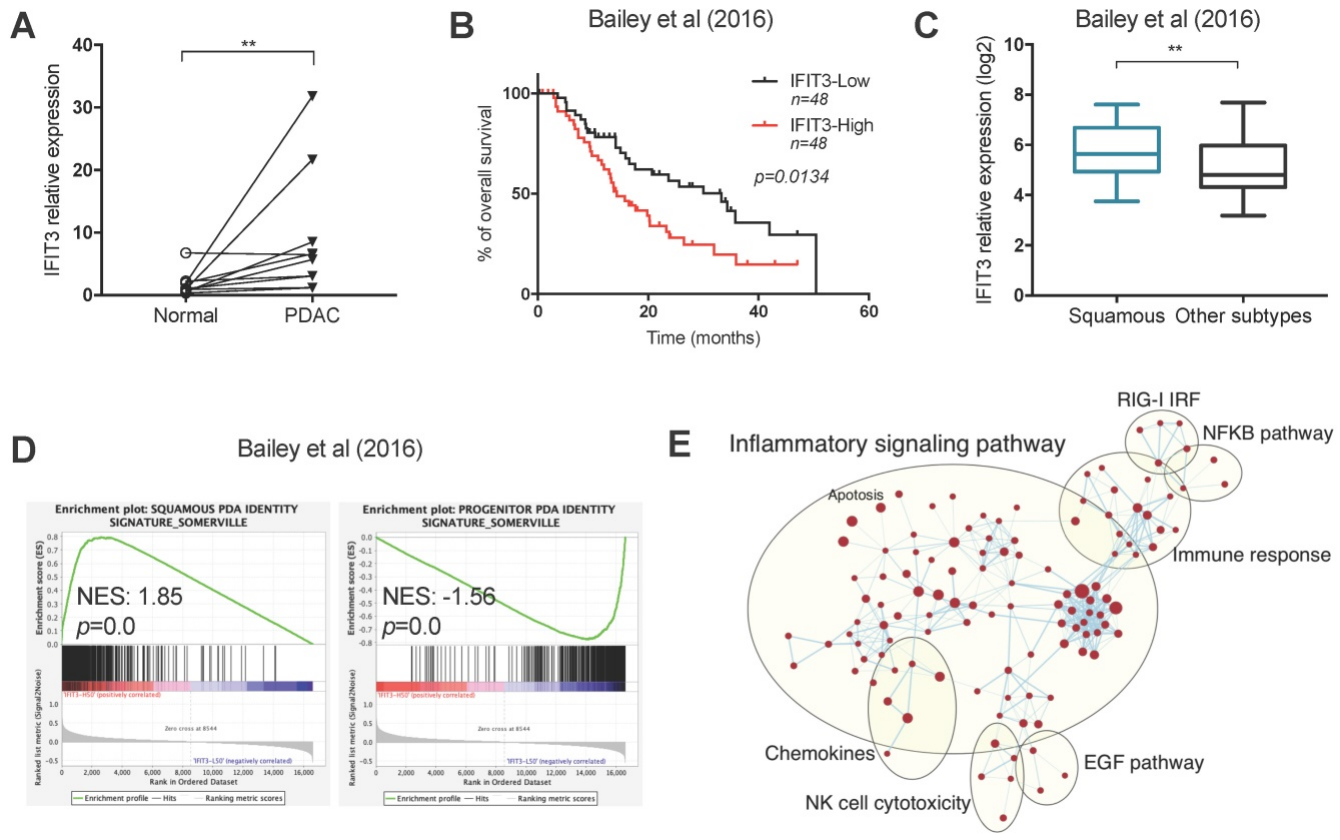
** Expression and characterization of IFIT3 in PDAC.** (A) IFIT3 expression is higher in PDAC tissues compare to adjacent normal tissues. Ten pairs of PDAC tissues and adjacent normal tissues were collected and analyzed with qRT-PCR. 18s rRNA was used as internal control. (B-E) Datasets from Bailey et al. were downloaded and analyzed. Samples were stratified into quantiles based on the expression of IFIT3 (lower 50% and upper 50% of values, n=48 for each group). (B) Kaplan-Meier survival analysis shows IFIT3 expression is associated with poor survival of PDAC patients. (C) IFIT3 expression is higher in squamous subtype of PDAC. Data are presented as box-and-whisker plot (Min to Max). (D) Gene set enrichment analysis shows enrichment of squamous signature in IFIT3-high group and progenitor signature in IFIT3-low group. (E) Enrichment map analysis shows IFIT3 expression is enriched with inflammatory response, immune response, NF-κB pathway and apoptosis. **p < 0.01.

To determine the *ex vivo* expression of IFIT3 in PDAC, 10 pairs of PDAC tissues and matched adjacent normal tissues were collected. qRT-PCR analysis showed that the expression of IFIT3 was higher in PDAC tissues as compared to adjacent normal tissues (Figure 1A). To further characterize the expression and potential function of IFIT3 in PDAC, RNA-sequence data from two PDAC cohorts were downloaded from cBioportal (QCMG, Bailey, Nature 2016; TCGA, PanCancer Atlas) [Supplementary file S1] and subjected to bioinformatics analysis 11,25. Survival data revealed that higher expression of IFIT3 was significantly associated with poor overall survival of PDAC patients, in both data sets (Figure 1B; Figure S1C). Using the dataset from Bailey et al 11, we found that IFIT3 was increased in the squamous subtype as compared to the other subtypes (Figure 1C). In addition, higher IFIT3 expression was associated with a higher stroma score and immune score in PDAC as seen in the Bailey dataset Figure S1A-B]. To characterize the potential function of IFIT3 in PDAC, a gene set enrichment analysis (GSEA) was applied to the datasets. In both datasets, the squamous signature as described by Bailey et al. was found to be enriched in IFIT3-high group, while the progenitor signature was found to be enriched in IFIT3-low group (Figure 1D; Figure S1D). Using enrichment map analysis, a series of molecular signatures were shown to be enriched in IFIT3-high group. These include inflammatory response, immune response, NF-κB pathway and apoptosis-related signatures (Figure 1E; Figure S1E). To address in more detail the association of IFIT3 with the squamous subtype of PDAC, a panel of PDAC cell lines were then examined. ∆Np63 was used as a marker for the squamous subtype [26. However, no correlation was found between the expression of IFIT3 and ∆Np63 in the PDAC cell lines examined (Figure S1F). By contrast, IFIT3 showed multiple roles in PDAC and thus may represent a robust marker to predict the treatment response in PDAC.

### IFIT3 interplays with NF-κB pathway during chemotherapy

**Figure 2:**
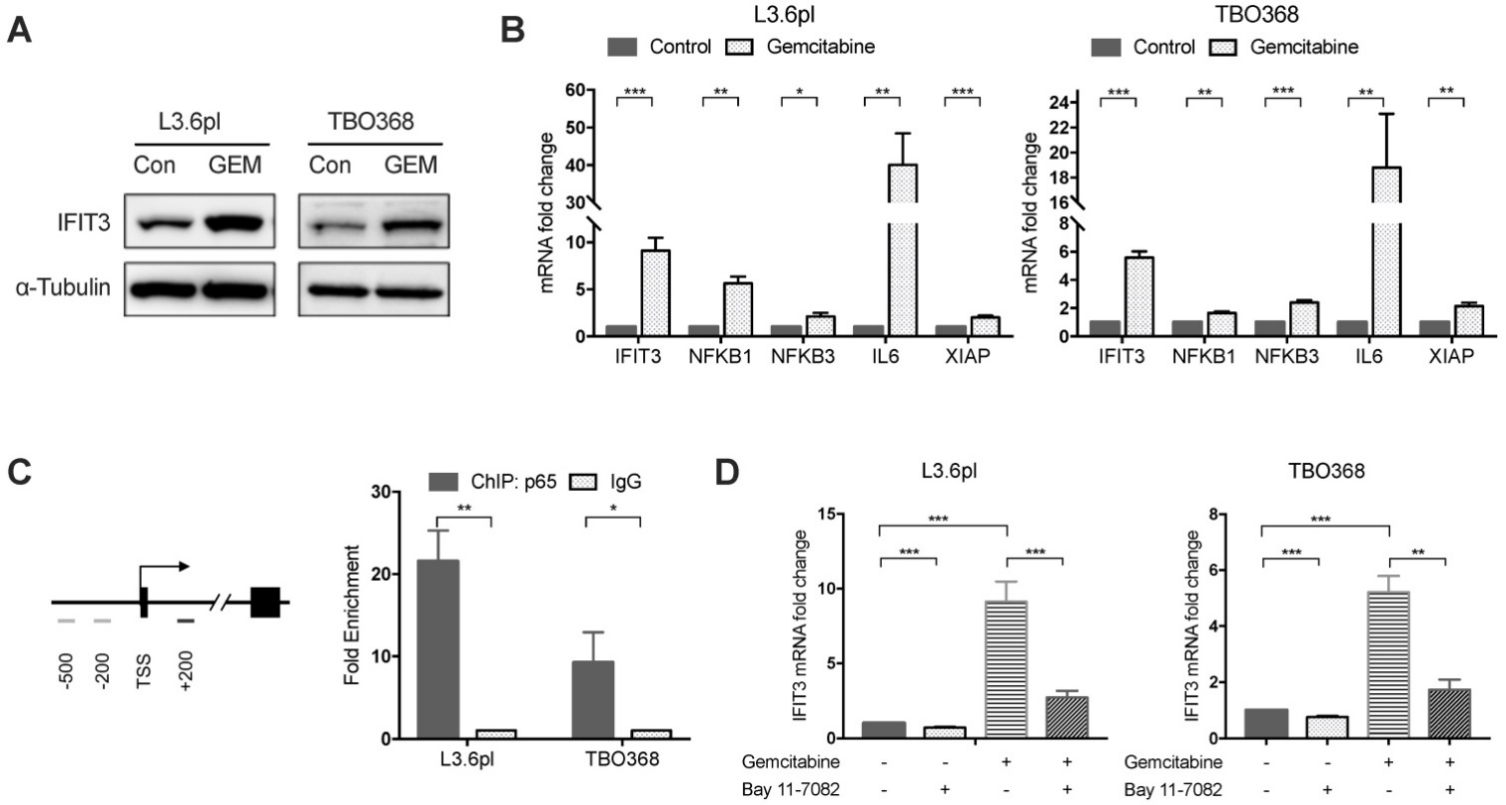
** IFIT3 interplays with NF-κB pathway during chemotherapy.** (A) Western blot showed IFIT3 was up-regulated after gemcitabine treatment. Protein samples were collected from L3.6pl and TBO368 cells with or without gemcitabine treatment (3 ng/ml for L3.6pl and 400 ng/ml for TBO368) for indicated time. Membrane was stripped after IFIT3 detection and re-probed with α-Tubulin. (B) IFIT3 and NF-kB pathway was activated after gemcitabine treatment in L3.6pl and TBO368. NFKB1, NFKB3, IL6 and XIAP were selected as indicators for activation of NF-κB pathway. RNA samples were collected from L3.6pl and TBO368 cells with or without gemcitabine treatment for indicated time. (C) Three putative p65 binding sites on IFIT3 gene promoter are depicted. Chromatin immunoprecipitation indicates direct binding of p65 to the promoter region of IFIT3 (TSS +122 to +342, black bar). Chromatin was extracted from 1% PFA fixed L3.6pl and TBO368 cells pre-treated with gemcitabine and immunoprecipitated with anti-p65 antibody or normal IgG. (D) Expression of IFIT3 was diminished by NF-κB inhibitor Bay 11-7082 with or without gemcitabine treatment. Bay 11-7082, 10 µM. Data are presented as mean ± SEM of three independent experiments. *p < 0.05, **p < 0.01, ***p < 0.001.

**Figure 3:**
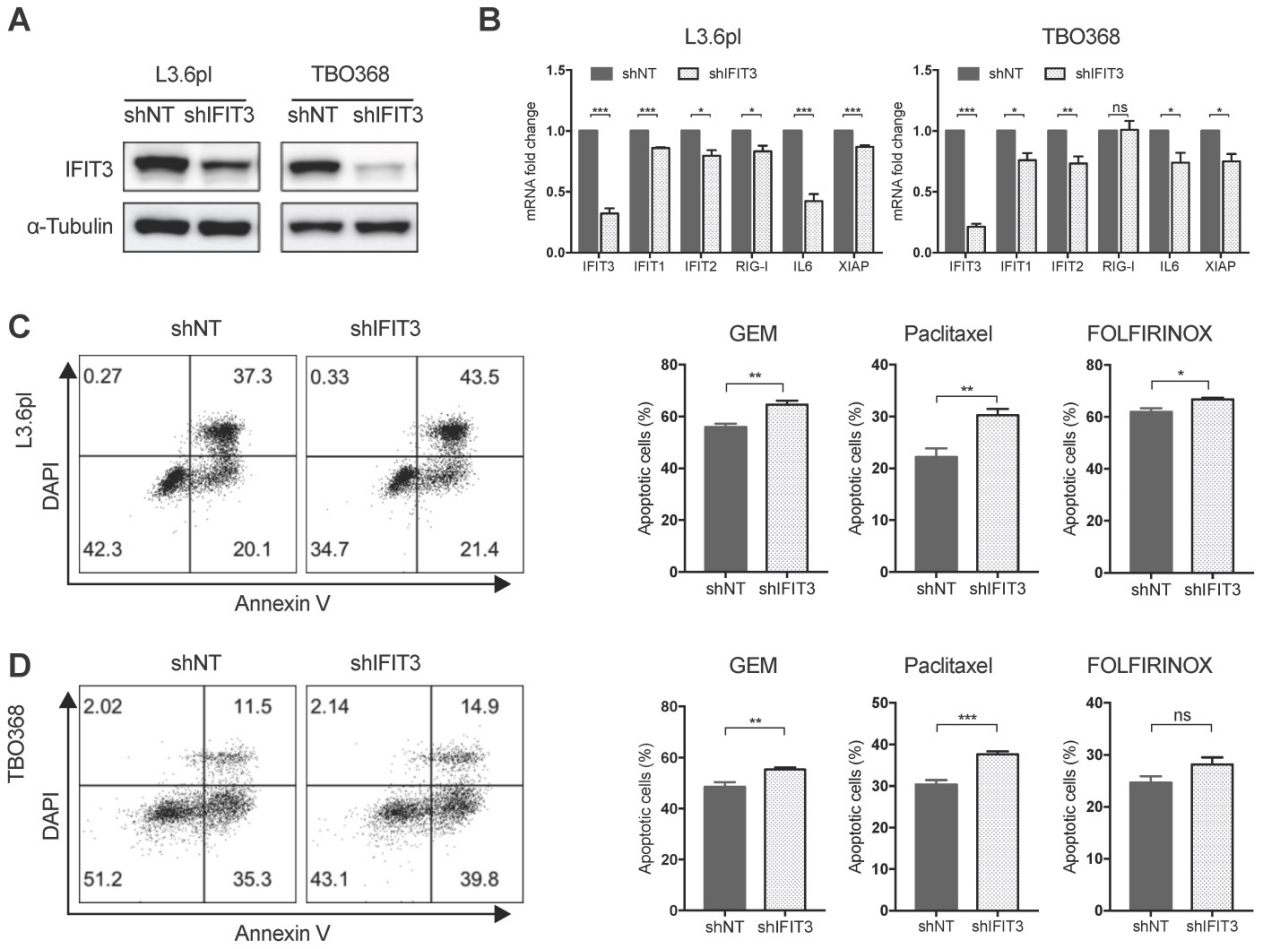
** IFIT3 renders chemotherapy resistance in PDAC cells.** (A) IFIT3 knockdown was confirmed with western blot in both L3.6pl and TBO368 cells. Membrane was stripped after IFIT3 detection and re-probed with α-Tubulin. (B) IFN pathway and NF-κB pathway targeted genes, IFIT1, IFIT2, RIG-I, IL6, and XIAP were significantly down-regulated after knockdown of IFIT3. (C-D) Knockdown of IFIT3 increased sensitivity of L3.6pl (C) and TBO368 (D) to chemotherapy. Cells were treated with gemcitabine (3 ng/ml for L3.6pl and 400 ng/ml for TBO368), paclitaxel (10 nM) or FOLFIRINOX (Oxaliplatin: Irinotecan: Folinic acid Calcium: 5-Fluorouracil=1:2:4:25 µM as 1X, used as 0.025X for L3.6pl and 0.5X for TBO368) for indicated time. Apoptosis was determined by flow cytometry analysis of Annexin V/DAPI staining. Representative FACS dot plots are shown on the left. Bar graphs are presented as mean ± SEM of three independent experiments. *p < 0.05, **p < 0.01, ***p < 0.001, ns: non-significant, *p* > 0.05.

Our previous work has shown that high expression of IFIT3 is associated with chemotherapy resistance in PDAC 20. To explore the role of IFIT3 in chemotherapy resistance, the expression of IFIT3 under chemotherapy was investigated. First, we observed the up-regulation of IFIT3 in our PDAC cell line L3.6pl and the patient-derived primary tumor cell line TBO368 after gemcitabine treatment at both the mRNA and protein level (Figure 2A-B). In addition, the NF-κB pathway was found to be activated in PDAC cells after gemcitabine treatment, as demonstrated by NFΚB1, NFΚB3 and the increase in the downstream target genes IL6 and XIAP (Figure 2B). As the NF-κB pathway signature was shown to be enriched in IFIT3-high group in PDACs, we hypothesized that NF-κB may be responsible for the up-regulation of IFIT3 during chemotherapy. Chromatin immunoprecipitation assay analysis showed binding of p65, a subunit of NF-κB, to the promoter region of IFIT3 (from TSS +122 to +341) (Figure 2C). To further verify the regulatory role of NF-κB in the expression of IFIT3, the effect of the specific NF-κB inhibitor Bay 11-7082 was studied. After Bay 11-7082 treatment the increase in IFIT3 was diminished with or without gemcitabine treatment (Figure 2D). These results suggest that NF-κB is involved in the up-regulation of IFIT3 in PDAC cells during chemotherapy, and that IFIT3 is a direct transcriptional target of NF-κB. IFIT3 knockdown cells were then established in L3.6pl and TBO368 (Figure 3A). Interestingly, we found that knockdown of IFIT3 diminished the expression of interferon pathway and NF-κB pathway genes, including IFIT1, IFIT2, RIG-I, IL6 and XIAP (Figure 3B). Taken together, the upexpression of IFIT3 seen during chemotherapy appears to be regulated in part by activation of the NF-κB pathway, and a positive feedback loop may exist between IFIT3 and NF-κB pathway.

### IFIT3 renders chemotherapy resistance in PDAC cells

To better characterize the role of IFIT3 in chemotherapy resistance of PDAC *in vitro*, IFIT3 knockdown and overexpression cell lines were established (Figure 3A; Figure S2A). Apoptosis assay analysis with flow cytometry and MTT assay were carried out to determine the impact of IFIT3 on chemotherapy response in the PDAC cells *in vitro*. Both L3.6pl and TBO368 IFIT3 knockdown cell lines showed more apoptosis upon gemcitabine treatment (Figure 3C-D; Figure S2B), while cells with IFIT3 overexpression showed less apoptosis as compared to control cells (Figure S2C-D). To identify a more general anti-apoptotic role of IFIT3 in the context of PDAC chemotherapy, the cytoskeletal drug paclitaxel and the FOLFIRINOX regimen were also applied in the same setting. Consistent with earlier results, knockdown of IFIT3 potentiated chemotherapy induced apoptosis by paclitaxel and FOLFIRINOX treatments (Figure 3C-D). These data show that IFIT3 renders chemotherapy resistance in PDAC cells independent of chemotherapy regimens.

### IFIT3 regulates the mitochondria-associated apoptosis

**Figure 4:**
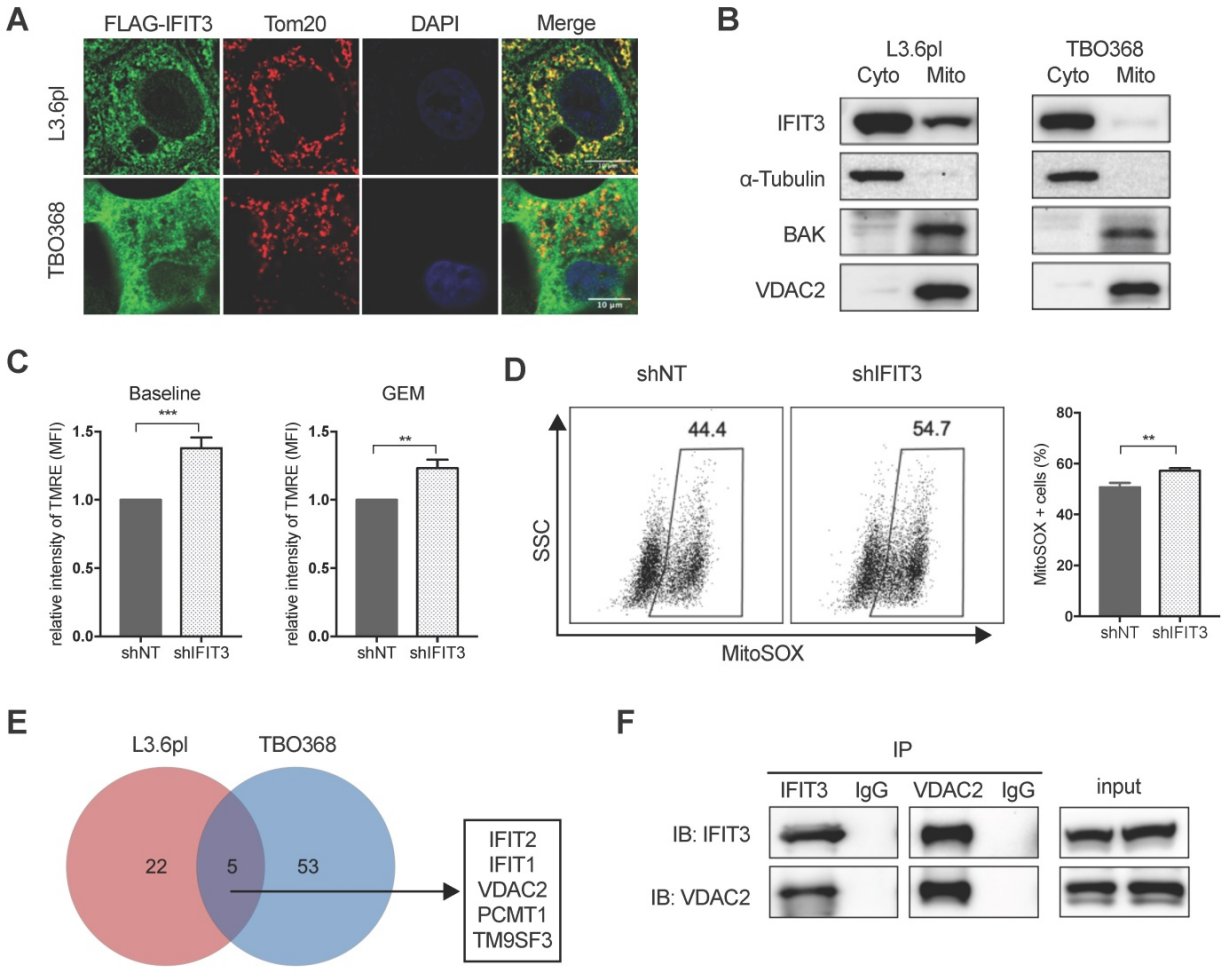
** IFIT3 regulates mitochondria-associated apoptosis.** (A) Confocal immunofluorescence labeled with anti-FLAG (*green*), anti-Tom20 (*red*), and counterstained with DAPI (*blue*), showed co-localization of FLAG-tagged IFIT3 and Tom20 in mitochondria. L3.6pl and TBO368 cells stably express FLAG-tagged IFIT3 were used here. Scale was shown in the lower-right corner. (B) Western blot showed localization of IFIT3 in cytosol and mitochondria of L3.6pl and TBO368. Mitochondria were isolated as indicated in methods section. Cyto represent cytosol and Mito represent mitochondria. We probed α-Tubulin as cytosolic marker, BAK and VDAC2 as mitochondrial marker. (C) IFIT3 knockdown in L3.6pl altered mitochondrial membrane potential (ΔΨm) with or without gemcitabine treatment. TMRE was used to determine the mitochondrial membrane potential (ΔΨm). MFI: mean fluorescence intensity. (D) IFIT3 knockdown in L3.6pl showed more MitoSOX positive cells when treated with gemcitabine. Representative FACS dot plot and bar graph are shown. (E) Mass spectrometry results of immunoprecipitated samples by anti-IFIT3 antibody are shown in Venn diagram. Cells were treated with gemcitabine for indicated time before harvest. Proteins with *p* < 0.01 and log2 difference > 1 are considered significant. Proteins identified in the intersection of L3.6pl and TBO368 are listed beside the diagram. (F) Interaction between IFIT3 and VDAC2 was confirmed with western blot in L3.6pl. Data are presented as mean ± SEM of three independent experiments. **p < 0.01, ***p < 0.001.

Mitochondria play essential role in the generation of reactive oxygen species (ROS) and cell apoptosis. To explore the possible role of IFIT3 in mitochondria-associated apoptosis, the localization of IFIT3 was examined using confocal immunofluorescence and subcellular fractionation. Immunofluorescence showed co-localization of IFIT3 with the mitochondrial outer membrane protein Tom20 (Figure 4A). However, western blot showed IFIT3 as predominantly a cytosolic protein, with a small fraction of IFIT3 localized to mitochondria (Figure 4B; Figure S3A). To investigate effects on ROS production in mitochondria, a mitochondrial membrane potential (ΔΨm) dye TMRE and mitochondrial superoxide indicator MitoSOX were used. The mitochondrial membrane potential (ΔΨm) was increased in IFIT3 knockdown cells as compared to control cells as indicated by TMRE staining, independent of gemcitabine treatment (Figure 4C). ROS production in mitochondria was significantly increased after gemcitabine treatment, while the IFIT3 knockdown cells showed enhanced MitoSOX as compared to control cells (Figure 4D). In addition, after knockdown of IFIT3, the anti-oxidative stress response gene ALDH1A3 was also downregulated (Figure S3B). Applying a quantitative cytochrome c release assay using flow cytometry showed more cytochrome c release from IFIT3 knockdown L3.6pl cells 27 (Figure S3C). In summary, these results suggest that a fraction of IFIT3 is localized in mitochondria of PDAC cells, and may play a role in the regulation of ROS production and mitochondria-associated apoptosis.

### IFIT3 interacts with the mitochondrial channel protein VDAC2

The results outlined above strongly suggest that IFIT3 renders chemotherapy resistance through the modulation of mitochondria-associated apoptosis. Protein-protein interaction (PPI) is an important characteristic of the tetratricopeptide repeat (TPR) found in IFIT3 28 (Figure S3D). The interaction of IFIT3 with proteins was then investigated to better understand the potential mechanism of IFIT3 mediated chemotherapy resistance in PDAC. Co-immunoprecipitation and mass spectrometry were performed to identify proteins interacting with IFIT3, with a special focus on mitochondrial proteins. A total of 80 IFIT3 interacting proteins were identified in L3.6pl and TBO368 cells (Figure 4E; Figure S3E). The proteins identified are listed in Supplementary file S2. GO analysis on this protein list was then performed. Biological function and process analysis showed an enrichment of proteins involved in response to virus, protein binding, protein transport and cellular protein localization (Supplementary file S3). However, only 5 proteins overlapped between both cell lines; IFIT1, IFIT2, VDAC2, PCMT1 and TM9SF3 (Figure 4E). Based on the results above linking IFIT3 to mitochondria and to further explore potential effects on mitochondrial-associated apoptosis, the voltage-dependent anion-selective channel 2 (VDAC2) was chosen for subsequent detailed analysis. As a first step, the interaction between IFIT3 and VDAC2 was confirmed by western blotting (Figure 4F).

### VDAC2 protects PDAC cells from chemotherapy induced apoptosis

**Figure 5:**
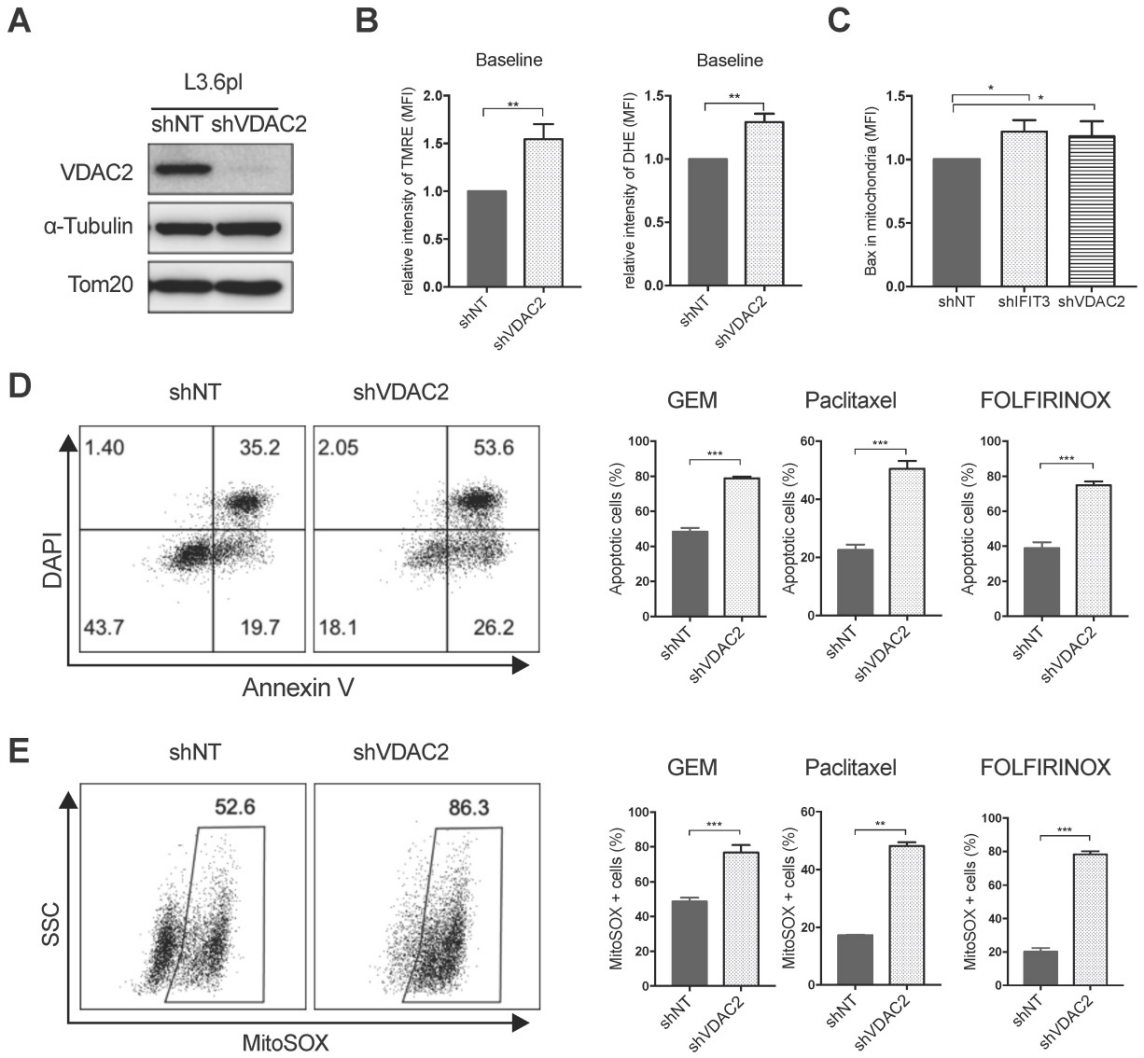
** VDAC2 protects PDAC cells from chemotherapy induced apoptosis.** (A) VDAC2 Knockdown was confirmed with western blot in L3.6pl. α-Tubulin and Tom20 were probed as loading control. (B) VDAC2 knockdown significantly increased the mitochondrial membrane potential (ΔΨm) and ROS production of L3.6pl in baseline. TMRE and DHE were used to determine the mitochondrial membrane potential (ΔΨm) and ROS production. (C) Translocation of BAX to mitochondria was increased after knockdown of IFIT3 and VDAC2 in L3.6pl. Cells were treated with gemcitabine for 48h before permeabilization and fixation. Samples were analyzed with flow cytometry. (D-E) VDAC2 knockdown increased sensitivity of L3.6pl to chemotherapy, as indicated by apoptotic assay (D) and MitoSOX staining (E). Representative FACS dot plot and bar graph are shown. Data are presented as mean ± SEM of three independent experiments. *p < 0.05, **p < 0.01, ***p < 0.001.

Using the PDAC tissue samples detailed in Materials and Methods, it was found that VDAC2 is also increased in PDAC tissues as compared to adjacent normal tissues (Figure S4A). However, survival analysis did not show a significant difference between the VDAC2-high and VDAC2-low groups (Figure S4B). Nevertheless, GSEA identified an enrichment of gene networks including Myc targets, DNA repair, oxidative phosphorylation and oxygen species pathway in the VDAC2-high group (Figure S4C-D). To further explore the role of VDAC2 in the regulation of chemotherapy induced apoptosis in PDAC, VDAC2 knockdown cells were generated (Figure 5A). The mitochondrial membrane potential (ΔΨm) and total ROS production were significantly increased after the knockdown of VDAC2 as indicated by TMRE and DHE staining (Figure 5B). Consistent with IFIT3 knockdown, VDAC2 knockdown significantly potentiated chemotherapy induced apoptosis, including gemcitabine, paclitaxel, and the FOLFIRINOX regimen (Figure 5D). ROS production in mitochondria during chemotherapy was also dramatically increased in the VDAC2 knockdown cells (Figure 5E). In addition, translocation of BAX to mitochondria during chemotherapy induced apoptosis was shown to be increased after the knockdown of IFIT3 or VDAC2 in PDAC cells (Figure 5C). Taken together, IFIT3 appears to regulate chemotherapy resistance in PDAC through the interaction with mitochondria outer membrane protein VDAC2, which functions as an anti-apoptotic protein in PDAC.

### IFIT3 modulates post-translational modification of VDAC2

**Figure 6:**
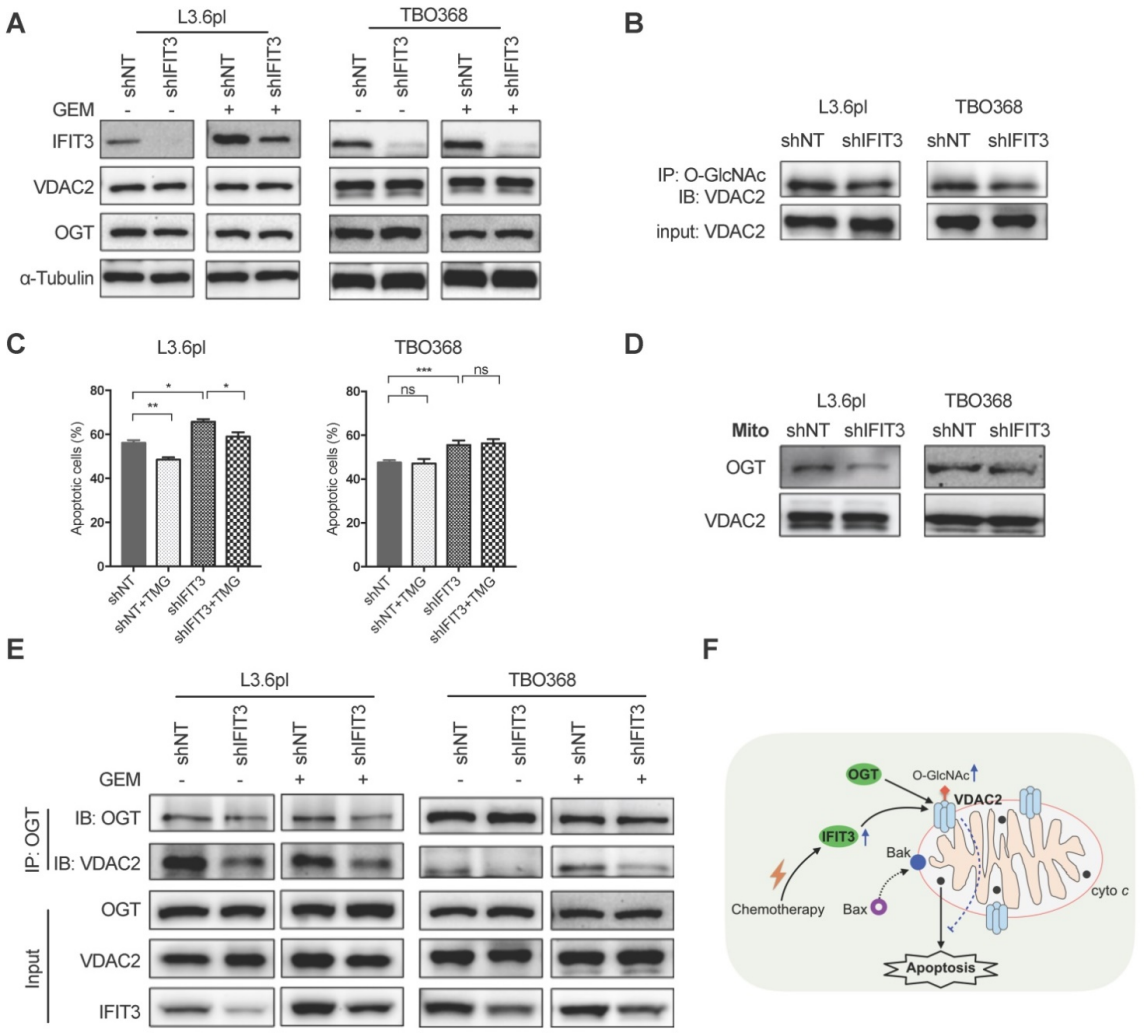
** IFIT3 modulates the O-GlcNAc level of VDAC2 through OGT.** (A) IFIT3 knockdown did not alter the protein level of VDAC2 in both L3.6pl and TBO368. Membranes were probed with IFIT3, VDAC2 and OGT, then stripped and re-probed with α-Tubulin as loading control. (B) IFIT3 knockdown decreased the O-GlcNAc modification level of VDAC2. O-GlcNAc modified proteins were immunoprecipitated with anti-O-GlcNAc (RL2) antibody and blot was probed with anti-VDAC2 antibody. Input of VDAC2 was probed for loading control. (C) TMG reduced gemcitabine induced apoptosis in L3.6pl while show no difference in TBO368. Cells were treated as indicated for 48 h and 72 h, in L3.6pl and TBO368, respectively. TMG, 5 µM. Data are presented as mean ± SEM of three independent experiments. (D) OGT level in mitochondrial fraction was decreased in IFIT3 knockdown cells compared to non-target control cells. Mitochondria were isolated as indicated in methods section. VDAC2 was used as loading control. (E) Immunoprecipitation of OGT showed less binding of VDAC2 after knockdown of IFIT3 in both L3.6pl and TBO368, with or without gemcitabine treatment. (E) Schematic picture of IFIT3 regulating the O-GlcNAc modification of VDAC2 and protecting PDAC cells from chemotherapy induced apoptosis. *p < 0.05, **p < 0.01, ***p < 0.001, ns: non-significant, *p* > 0.05.

To gain deeper understanding of the interaction between IFIT3 and VDAC2, the impact of IFIT3 expression on the function of VDAC2 was further evaluated. As a first step, the expression of VDAC2 was evaluated in IFIT3 knockdown and overexpression cells. Neither the steady state mRNA level (data not shown) nor the protein level of VDAC2 was affected by IFIT3 knockdown or overexpression (Figure 6A; Figure S5E). Thus, we hypothesized that the impact of IFIT3 on VDAC2 may appear in post-translational modification (PTM) of the protein. VDAC2 has been reported to undergo phosphorylation, acetylation, tyrosine nitration, and O-GlcNAcylation modifications 29. Though not significant, we observed a slight decrease in total O-GlcNAc levels in IFIT3 knockdown cells (Figure S5A). To evaluate whether IFIT3 could directly impact the O-GlcNAc modification of VDAC2, immunoprecipitation was performed using anti-O-GlcNAc antibody. The western blot results show that the level of O-GlcNAc modification of VDAC2 was decreased in IFIT3 knockdown cells, and increased in IFIT3 overexpression cells (Figure 6B; Figure S5E). The dynamic regulation of O-GlcNAc was controlled by a pair of enzymes, O-GlcNAc transferase (OGT) and O-GlcNAcase (OGA). To rule out a non-specific blotting of O-GlcNAc, an OGA inhibitor Thiamet G (TMG) and OGT inhibitor OSMI-1 were used. As shown in Figure S5B, TMG significantly increased total O-GlcNAc levels while OSMI-1 decreased O-GlcNAc levels in PDAC cells. More importantly, TMG increased the O-GlcNAc level of VDAC2, while OSMI-1 did the opposite (Figure S5C). To test the effect of O-GlcNAc modification on chemotherapy induced apoptosis in PDAC cells, TMG or OSMI-1 was added during chemotherapy. A protecting role of O-GlcNAc modification in PDAC during chemotherapy was observed (Figure 6C; Figure S5D).

As O-GlcNAc is directly added by OGT in cells 30, the involvement of OGT in the regulation of O-GlcNAc modification of VDAC2 was investigated. The protein level of OGT was not changed in IFIT3 knockdown or overexpression cells when compared to control cells (Figure 6A; Figure S5E). When examined at the subcellular level, however, IFIT3 expression had an effect on the enrichment of OGT in mitochondria (Figure 6D; Figure S5F). Furthermore, in immunoprecipitation studies OGT showed less binding to VDAC2 in IFIT3 knockdown cells (Figure 6E). More VDAC2 was found to be bound to OGT in IFIT3 overexpression cells (Figure S5E). Taken together, these results show that IFIT3 may modulate the O-GlcNAc level of VDAC2 via the recruitment or stabilization of the interaction between OGT and VDAC2, thus, increasing the O-GlcNAc level of VDAC2, and protecting PDAC cells from chemotherapy induced apoptosis.

## Discussion

Chemotherapy represents a major treatment option to prolong the survival of PDAC patients, but chemotherapy resistance emerges over time 2. Recently, two molecular subtypes of PDAC have been described that are referred to as classical and basal-like subtypes 10,11,31. The basal-like subtype, also sometimes called the squamous subtype or quasi-mesenchymal subtype, has a poor PDAC prognosis 10,11,31-33. The enriched gene networks identified in the squamous subtype include; inflammation, hypoxia response and metabolic reprogramming, may help explain the resistance to treatment seen in these PDAC patients.

The characterization of specific molecular signatures can be used to better understand the drug response in PDAC. By using the datasets described by Bailey et al. 11 and the TCGA database 25, we could show that an enhanced expression of IFIT3 was linked to the squamous subtype of PDAC. The subsequent analysis of a panel of PDAC cell lines did not show a strong correlation of IFIT3 with ∆Np63, a marker previously reported to identify the squamous subtype of PDAC 26. Importantly, Moffitt et al. reported that all the PDAC cell lines tested in their study were found to represent only the basal-like subtype (corresponding to squamous subtype) 31. The database-derived signature may not be well represented by *in vitro* cultured cell lines.

Our previous work had suggested a possible role of IFIT3 in chemotherapy resistance of PDAC 20. The transcriptomic analysis identified enriched pathway signatures for inflammatory response, immune response, NF-κB pathway and apoptosis in the IFIT3-high patient group, suggesting points at which IFIT3 may influence the treatment response of PDAC. Among the enriched gene networks identified, the NF-κB pathway had been previously shown to play an important role in chemotherapy resistance in PDAC 9,34. PDAC cells have been shown to gain protection against apoptosis through an overexpression of Bcl2 family proteins, or activation of the NF-κB pathway, that results in the activation of target genes such as the endogenous inhibitor of caspase function XIAP. In the present study, we observed an up-regulation of both IFIT3 and NF-κB pathway in response to chemotherapy. Previous investigations have revealed that IFIT3 is a direct transcriptional target of the NF-κB pathway, and that knockdown of IFIT3 decreased the expression of other NF-κB targeted genes (including IL6 and XIAP) suggesting a positive feedback loop for IFIT3. Liu et al. also reported that IFIT3 acts as a positive regulator of RIG-I signaling, and that exogenous expression of IFIT3 enhanced activation of the NF-κB pathway 15. Crosstalk between the interferon and NF-κB pathway has been proposed 35. Thus, at least to some extent, IFIT3 may influence the chemotherapy response through effects on NF-κB pathway activation.

In addition to an effect of IFIT3 on NF-κB pathway signaling, we show here that the influence of IFIT3 also extends to mitochondria function which further contributes to chemotherapy resistance in PDAC. Mitochondria represent the main energy source for cells, but they also help control cell fate in response to the oxidative stress brought about by chemotherapy. During mitochondria-associated apoptosis, the formation of mitochondrial permeability transition pores and the subsequent release of mitochondrial intermembrane proteins, like cytochrome c, result in the activation of caspases and eventually cell death 36. In our study, we found IFIT3 renders chemotherapy resistance in PDAC in line with the reported role of IFIT3 as an anti-apoptotic factor 37,38. Furthermore, we could demonstrate that a portion of IFIT3 was localized to mitochondria and that knockdown of IFIT3 lead to enhanced ROS production by mitochondria. However, the molecular mechanism involved here is at present unknown. qRT-PCR analysis did show a down regulation of the antioxidant protein ALDH1A3 in IFIT3 knockdown cells. ALDH1A3 has been reported to be a cancer stem cell marker in breast cancer 39. These observations support a potential role for IFIT3 in redox regulation and mitochondria-associated apoptosis.

Direct protein-protein interaction is an important feature of IFIT proteins 28. We speculated that IFIT3 may render chemotherapy resistance in PDAC cells through these protein-protein interactions with mitochondrial proteins. Using immunoprecipitation and mass spectrometry, we were able to identify VDAC2 as a potential interacting protein with IFIT3. VDAC2 is a channel protein found at the outer mitochondrial membrane that controls the transportation of metabolites across the mitochondrial outer membrane, and has been shown to regulate ROS production in mitochondria 21. These actions of VDAC2 in regulating energy metabolism and mitochondria-associated apoptosis makes VDAC2 an interesting potential target for cancer treatment 21. A potential role for VDAC2 in moderating chemotherapy resistance is intriguing, and both pro-apoptotic 40,41 and anti-apoptotic 23,42 actions of VDAC2 have been reported.

The role of VDAC2 in PDAC is not well understood. We show here that knockdown of VDAC2 sensitized PDAC cells to the chemotherapy response that is independent of chemotherapy regimen. Our results strongly suggest that the regulation of ROS production and mitochondria-associated apoptosis may be mediated by IFIT3 through its interaction with VDAC2.

Importantly, in this context, the knockdown of IFIT3 did not alter the steady state mRNA or protein levels of VDAC2. Post-translational modifications have been suggested as a means to regulate the diverse physiological functions identified for VDAC2 29. Various post-translational modifications of VDAC2 have been reported, including phosphorylation, acetylation, and O-GlcNAcylation 29,43,44. For example, Das et al. showed that GSK-3β mediated phosphorylation of VDAC2 promoted apoptosis during myocardial ischemia/reperfusion injury 45. However, the effect of the various PTMs especially O-GlcNAcylation on VDAC2 function have not been widely reported.

An increased O-GlcNAcylation in cancer cells is thought to be associated with chemotherapy resistance 46. High O-GlcNAc modification has been found in pancreatic cancer, breast cancer, lung and colon cancer, hepatocellular carcinoma, and prostate cancer 47. In PDAC, a higher level of O-GlcNAc was associated with constitutive activation of the NF-κB pathway and chemotherapy resistance 48,49. O-GlcNAcylation of VDAC2 has been reported in several proteomics based studies; however, the functional effect of this modification on VDAC2 is not understood. Palaniappan et al. reported that a global O-GlcNAc perturbation induced mitochondria dysfunction and apoptosis through VDAC2 in MEF cells 50. However, the results shown here suggest that higher O-GlcNAc levels protect PDAC cells from chemotherapy induced apoptosis, and that IFIT3 plays important role in regulating the O-GlcNAc modification of VDAC2. We observed that knockdown of IFIT3 decreased the O-GlcNAc level of VDAC2, while overexpression of IFIT3 increased it.

O-GlcNAcylation is a highly dynamic PTM. The O-GlcNAc group is added by O-GlcNAc transferase** (**OGT) and removed by O-GlcNAcase (OGA) in cells 30. The involvement of OGT in the regulation of O-GlcNAc modification of VDAC2 in IFIT3 knockdown cells was investigated. Using the proteomic data published by Gao et al, we could identify binding of OGT to VDAC2 and other mitochondria proteins 51. Our results further demonstrated that IFIT3 affected the subcellular distribution of OGT in mitochondria and that IFIT3 increased binding of OGT to VDAC2. GO analysis also revealed an enrichment of proteins involved in cellular protein translocation. Thus, IFIT3 may act in part through the recruitment or stabilization of interaction of VDAC2 with OGT. In this regard, IFIT3 may act as an intermediary regulator, by promoting the O-GlcNAc modification of VDAC2 via OGT.

It is at unknown if these IFIT3 actions are specific to the squamous subtype of PDAC, or may be relevant for other types of tumors. As discussed earlier, all of the *in vitro* cultured cell lines represent the squamous subtype of PDAC 31. A more physiologic model system, such as the organoid approach, may be required to investigate this issue in more detail. To this end, a high expression of IFIT3 predicting poor survival could only be found in TCGA-LAML, TCGA-PAAD and TCGA-THYM using the GEPIA web tool. In TCGA-KIRC, TCGA-MESO, TCGA-SARC and TCGA-SKCM, higher IFIT3 expression level was associated with better outcome (data not shown). Based on these early observations, it appears that IFIT3 may not act the same way in other tumors. Importantly, one can also not conclude that IFIT3 exclusively impacts VDAC2 O-GlcNAcylation and chemoresistance in PDAC, or even that it is restricted to squamous PDAC.

An enrichment of immune response networks was found in the IFIT3 high PDAC patient group. Using the PDAC tissue data reported by Bailey, we could show that higher IFIT3 expression is associated with higher immune score including; an enhanced macrophage signature, CD4+ regulatory T cell signature and APC co-inhibition signature. Immunotherapy represents a new frontier for cancer therapy. The check point inhibitors that have shown such promise in other tumor settings have failed to improve survival of PDAC patient groups 52. This failure of immunotherapy in PDAC is thought to be due to low immunogenicity, a largely non-inflamed phenotype and the unique desmoplastic stroma present 53. Exogenous expression of IFIT3 can be associated with increased desmoplasia as shown in our previous work 54. Targeting IFIT3 could thus also provide an option to enhance the effectiveness of immunotherapy in PDAC. This is an interesting and relevant hypothesis that needs to be tested.

In conclusion, based on the findings shown here, we propose that IFIT3 may act as a reliable molecular marker to predict the chemotherapy response in PDAC. Our results further demonstrate that a NF-κB triggered up-regulation of IFIT3 may enhance chemotherapy resistance in PDAC cells through modulation of O-GlcNAc modification of VDAC2. Targeting IFIT3/VDAC2 may represent a potential strategy to re-sensitize aggressive pancreatic cancer to conventional chemotherapy regimens.

## Methods and Materials

### Antibodies and Reagents

Mouse monoclonal anti-α-Tubulin (Cell signaling, 3873), rabbit polyclonal anti-IFIT3 (Invitrogen, PA5-22230), mouse polyclonal anti-IFIT3 (Abcam, ab76818), rabbit polyclonal anti-VDAC2 (Invitrogen, PA5-28106), mouse monoclonal anti-FLAG (Invitrogen, MA1-91878), rabbit monoclonal anti-OGT (Cell signaling, 24083), mouse monoclonal anti-O-GlcNAc (Clone, RL2) (Biolegend, 677902), mouse monoclonal anti-cytochrome c (Biolegend, 612302), rabbit monoclonal anti-Bak (Cell signaling, 12105), rabbit monoclonal anti-Bax (Cell signaling, 5023), rabbit monoclonal anti-FASN (Cell signaling, 3180), rabbit polyclonal anti-Tom20 (Sigma-Aldrich, HPA011562), HRP-conjugated secondary antibody (Invitrogen, 31430 and 31460); NF-κB inhibitor Bay 11-7082 (Cayman, 10010266), OGA inhibitor Thiamet G (Medchemexpress, HY-12588), OGT inhibitor OSMI-1 (Abcam, ab235455) were purchased from the indicated manufacturers. Chemotherapeutic agents including gemcitabine (Gemcitabin Hexal, Hexal AG), paclitaxel (NeoTaxan, Hexal AG) and FOLFIRINOX (Oxaliplatin, Accord HealthCare; Irinotecan, Amneal Deutschland GmbH; Folinic acid Calcium, Lyomark Pharma GmbH; 5-Fluorouracil, Accord HealthCare) were supplied by University Hospital Cologne.

### Cell culture and human materials

Human pancreatic adenocarcinoma cells L3.6pl were cultured in DMEM (Gibco, USA) supplemented with 10% FBS, 1% vitamin mixture, 1% NEAA, 2mM L-glutamine and 1% penicillin/streptomycin as previously described 55. PDAC patient derived primary cells TBO368 were cultured in advanced DMEM (Gibco, USA) supplemented with 10% FBS, 2mM L-glutamine and 1% penicillin/streptomycin as previously described 56. HEK293T, MIA PaCa-2, PANC-1, HPAFII, BxPC3, Capan-2 and SW1990 were cultured in DMEM (Gibco, USA) supplemented with 10% FBS, 2mM L-glutamine and 1% penicillin/streptomycin. All cell lines were tested for mycoplasma contamination. PDAC tissues and adjacent normal tissues were ethically collected from biobank under approval of BIOMASOTA (approved by the Ethics Committee of the University of Cologne, ID: 13-091).

### Quantitative Real-time PCR (qRT-PCR)

RNA was extracted from cultured cells or tissue samples with TRI reagent (Sigma-Aldrich). For reverse transcription, High-Capacity cDNA Reverse Transcription Kit (Applied Biosystems, Thermo Fisher Scientific, USA) was used according to the manufacturer's instruction. Target mRNAs were determined using the Fast SYBR green master mix (Invitrogen) with QuantStudio 7 flex (Applied Biosystems, Thermo Fisher Scientific, USA). Primers were listed in Table S1.

### Isolation of mitochondria

Cells were harvested and washed in PBS, pelleted at 370g for 5 min, resuspended in homogenization buffer (20 mM HEPES, pH 7.2, 250 mM sucrose, 10 mM KCl, 0.15 mM MgCl2) supplemented with proteinase inhibitors. Cells were homogenized by passing through a 26G needle fitted on a 1 mL syringe, 60-80 passages were required. Then intact cells and debris were pelleted at 1000× g for 5 min, the supernatant was transferred to a new tube. This step was repeated once more. The supernatant was centrifuged at 8000× g for 10 min. The supernatant was collected as cytosolic fraction. The pellet was washed once with homogenization buffer and signed as mitochondria fraction. Samples were boiled in 1x LDS sample buffer at 70 degree for 10 min and subjected to SDS-PAGE.

### Western blot

Cells were harvested and lysed with RIPA buffer (Cell signaling, 9806) in ice for 30 min and centrifuged at 12000× g for 10 min. Supernatant was collected and protein concentration was measured using BCA method (Thermo Fisher Scientific). Protein samples were boiled in 1x NuPAGE LDS sample buffer (Invitrogen) at 70℃ for 10 min. Twenty microgram protein samples were subjected to 7.5% - 15% gradient SDS-PAGE gel and transferred to PVDF membrane (MACHEREY-NAGEL, Germany). The membranes were blocked in 1x Roti-Block (Carlroth, Germany) at room temperature for 1 h and were then probed with specific primary antibodies overnight at 4°C. Blots were incubated with HRP-conjugated secondary antibody (Invitrogen, 31430 and 31460) for 1 h at room temperature. Bands were visualized by SuperSignal West Pico PLUS Chemiluminescent Substrate (Thermo Fisher Scientific, USA) and detected using the ChemoStar ECL Imager (Intas Science Imaging, Germany).

### MTT assay

Cells were seeded into 96-well plate overnight and then treated with drugs for 48-72 h. Cell viability was detected by MTT assay. Briefly, after treatment, cells were incubated with MTT at 37°C for 3 hours. Then medium was discarded and MTT solvent was added. Absorbance at 570 nm was measured with a plate reader. All assays were performed in triplicate.

### Flow cytometry analysis

L3.6pl and TBO368 cells were treated with gemcitabine, paclitaxel, or FOLFIRINOX for 48 h and 72 h, respectively. For apoptosis analysis, cells were harvested and resuspended in Annexin V binding buffer, with Annexin V (Biolegend) and DAPI staining dye, incubated at room temperature for 20 min. For detection of mitochondrial membrane potential and ROS production, cells were stained with tetramethylrhodamine, ethyl ester (TMRE), dihydroethidium (DHE), or MitoSOX (Thermo Fisher Scientific) at room temperature for 20 min, respectively. For quantitative cytochrome *c* release assay, samples were prepared as described previously in literature with some modification 27. Briefly, cells were harvested and resuspended in permeabilization buffer (PBS, pH 7.4, 100 mM KCl, 2.5 mM MgCl2) containing 0.01% saponin, incubated on ice for 5 min, until >95% cells were permeabilized (confirmed with trypan blue uptake). Cells were fixed in 4% paraformaldehyde for 20 min and further permeabilized with 0.1% saponin in PBS and blocked for 30 min. Cells were incubated overnight at 4 ℃ with anti-cytochrome c antibody (Biolegend, clone 6H2.B4), washed and incubated 1 h at room temperature with Alexa Fluro 568 labeled secondary antibody (Invitrogen). Intracellular Bax staining was prepared as above with anti-Bax antibody (Cell signaling, 5023). Samples were subjected to analysis on CytoFLEX cytometer. Data were analyzed with Flowjo software (Tree star, Ashland, USA).

### Plasmid constructs

For expression of FLAG-tagged IFIT3, FLAG-IFIT3 vector was purchased from Vectorbuilder (China). For RNA interference, shRNA sequences for IFIT3 and VDAC2 were synthesized (Thermo Fisher Scientific) and inserted via AgeI and EcoRI into the Tet-pLKO-puro vector (Addgene, 21915). shRNA target sequences were: non-target control, 5'-AGGTAGTGTAATCGCCTTGTT-3'; IFIT3, 5'-GCTATGGACTATTCGAATAAA-3'; VDAC2, 5'-GCAAAGCTGCCAGAGATATTT-3'. All expression vectors were confirmed by DNA sequencing.

### Lentiviral transduction

Cells stably expressing shRNA sequence were created by lentiviral transduction. Briefly, HEK293T cells were co-transfected with transfer vector and packaging vectors (Addgene) using PEI (Sigma-Aldrich) in a mass ratio of 1:3 of DNA/PEI. Medium was changed 24 h later, and virus containing supernatant were collected and filtered at 48 h and 72 h. Virus containing supernatant were mixed 1:1 with fresh medium, supplemented with 8 µg/ml polybrene, and used to transduce cells. Puromycin was added 48-72 h later and selective medium was changed every 2 days and maintained for 1 week. shRNA expression was induced with 1 µg/ml Doxycycline (Sigma-Aldrich).

### Immunoprecipitation and mass spectrometry

For immunoprecipitation, cells were lysed with IP lysis buffer (25 mM Tris, pH 7.4, 150 mM NaCl, 1mM EDTA, 0.5% NP-40, 5% glycerol) and sonicated briefly. Supernatant was collected after centrifugation at 4°C, 12000× g, 15 min. Then protein samples were subjected to indicated antibody or normal IgG control at 4°C overnight. Then protein-antibody complexes were precipitated with protein A-Dynabeads (Invitrogen) and eluted with 1x LDS sample buffer at 70°C for 10 min. Eluted proteins were whether subjected to SDS-PAGE or mass spectrometry (MS). For mass spectrometry, samples were processed with in-gel digestion. Briefly, eluted proteins were reduced and alkylated with DTT (5mM) and CAA (40mM), respectively. Samples were loaded onto a 10% acrylamide gel, and run with constant voltage till the samples were into the stacking gel. Each lane of gel was cut into small pieces (~1mm per side) and immerged into 100 µL 50 mM ABC/50% ACN. Gel pieces were then dehydrated by incubation with 100 µL Acetonitrile and dried in the Speedvac. Then gel pieces were destained and dehydrated by incubation with 50 mM ABC and Acetonitrile, sequentially. This step was repeated until the gel was destained and then dried in the Speedvac. Then digestion solution with 10 ng/µL of 90% Trypsin and 10% LysC in 50 mM ABC was added to cover the gel pieces and incubated in ice for 30 min for swelling. Excess digestion solution was removed and 50 mM ABC was added to cover the gel pieces completely and incubated at 37°C overnight. Digested peptides in gel pieces were eluted using 30% ACN / 3% TFA and 100% ACN, sequentially. Supernatants were collected from each steps and combined, then concentrated in Speedvac to a final volume of approximately < 50µL. Samples were acidified by addition of formic acid to a final concentration of 1% and then purified with SDB RP StageTip. Nano LC-MS was performed and analyzed using the MAXQuant (Max Planck Institute of Biochemistry, Martinsried, Germany) and Perseus software (Max Planck Institute of Biochemistry, Martinsried, Germany).

### Chromatin immunoprecipitation

For chromatin immunoprecipitation, cells were fixed with 1% formaldehyde at room temperature for 12 min and neutralized with glycine (125 mM) for 3 min. Cells were washed with ice cold PBS for 2 times, then scraped and pelleted. Cell pellets were resuspended in pre-lysis buffer (20mM Tris, 150mM NaCl, 1mM EDTA, 1% Triton X-100, 0.1% SDS, pH 7.5) for eliminating cytosol protein contamination. Cells were pelleted again, resuspended in SDS lysis buffer (50 mM Tris, 10 mM EDTA, 1% SDS, pH 8). Chromatin was sheared and diluted in ChIP dilution buffer (20 mM Tris-HCl, 150 mM NaCl, 1% Triton X-100, 1 mM EDTA, pH 7.5). Then chromatin samples were subjected to anti-p65 (Cell signaling, 8252) or normal IgG control (Cell signaling, 2729) at 4℃ overnight. Then protein-antibody complexes were precipitated with protein A-Dynabeads (Invitrogen). Immunoprecipitated complexes were washed and eluted with buffer (1% SDS, 0.1 M NaHCO_3_) and then incubated with Proteinase K for 4 h at 65 °C on a thermomixer. DNA was purified using the PCR Clean Up Kit (MACHEREY-NAGEL, Germany) and subjected to quantitative PCR for IFIT3 promoter detection. Used primers were: IFIT3-for, 5'-CTGATGCGTGCCCTACTCTC-3'; IFIT3-rev, 5'-GGAATGTGCCTGCACAGTAAG-3'.

### RNA-seq data analysis and survival analysis

Pre-processed RNA-seq data and matched clinical data were downloaded from cBioportal (QCMG, Bailey, Nature 2016; TCGA, PancCancer Atlas). Gene set enrichment analysis was performed using GSEA software and enrichment map analysis was performed using Cytoscape. The sample GSVA score of different immune signature was extracted from Bailey's article 11. Survival analysis was performed using the Kaplan-Meier method and the difference was tested with log-rank test. *p* value < 0.05 was considered statistically significant.

### Statistical analyses

Statistical analysis was done using GraphPad Prism 7. Data were presented as mean ± SEM. Statistical significance was determined by two-sided unpaired *t*-test. *p < 0.05, **p < 0.01, ***p < 0.001, ns: non-significant, *p* > 0.05.

## Abbreviations

ChIP: Chromatin immunoprecipitation
DHE: Dihydroethidium
FOLFIRINOX: FOL folinic acid
F: fluorouracil
Irin: irinotecan
Ox: oxaliplatin
GSAE: Gene set enrichment analysis
IFIT3: Interferon-induced protein with tetratricopeptide repeats 3
OGA: O-GlcNAcase
OGT: O-GlcNAc transferase
O-GlcNAc: O-linked beta-N-acetylglucosamine
OSCC: oral squamous cell carcinoma
PDAC: Pancreatic ductal adenocarcinoma
PTMs: Post-translational modifications
ROS: Reactive oxygen species
TMRE: Tetramethylrhodamine ethyl ester
TPR: Tetratricopeptide repeat
VDAC2: Voltage-dependent anion-selective channel protein 2
XIAP: X-Linked Inhibitor of Apoptosis
