# Supplementary material for: Inflammatory IFIT3 renders chemotherapy resistance by regulating post-translational modification of VDAC2 in pancreatic cancer
Source: Theranostics. 2020 Jun 1;10(16):7178–92. doi: 10.7150/thno.43093 (PMC7330856; doi:10.7150/thno.43093)
Supplement: Supplementary file 1 — Supplementary figures and tables. [file thnov10p7178s1.pdf]

Supplemental information

Inflammatory IFIT3 renders chemotherapy resistance by regulating post-translational modification of VDAC2 in pancreatic cancer

Zhefang Wang[1], Jie Qin[1], Jiangang Zhao[1,2], Jiahui Li[1], Dai Li[1,3], Marie Popp[1], Felix Popp[1], Hakan Alakus[1], Bo Kong[4], Qiongzhu Dong[5], Peter J. Nelson[6], Yue Zhao[1], Christiane J. Bruns[1]

1. Department of General, Visceral und Tumor Surgery, University Hospital Cologne, Kerpener Straße 62, 50937 Cologne, Germany; zhefang.wang@uk-koeln.de; jie.qin@uk-koeln.de; jiangang.zhao@uk-koeln.de; jiahui.li@uk-koeln.de; marie.popp@uk-koeln.de; felix.popp@uk-koeln.de; hakan.alakus@uk-koeln.de; yue.zhao@uk-koeln.de; christiane.bruns@uk-koeln.de.
2. Department of General, Visceral und Vascular Surgery, Ludwig-Maximilian-University (LMU), 81377 Munich, Germany.
3. Department of Anesthesiology, Changhai Hospital, Naval Medical University, Shanghai, PR China; lidaichhosp@163.com.
4. Department of Surgery, Klinikum Rechts der Isar, Technische Universität München, Munich, Germany. kongbo81@hotmail.com.
5. Department of General Surgery, Huashan Hospital & Cancer Metastasis Institute & Institutes of Biomedical Sciences, Fudan University, Shanghai, China. qzhdong@fudan.edu.cn.
6. Medizinische Klinik und Poliklinik IV, University of Munich, Munich, Germany. peter.nelson@med.uni-muenchen.de.

* Correspondence: yue.zhao@uk-koeln.de, christiane.bruns@uk-koeln.de; Tel.: +49 221 478 30601

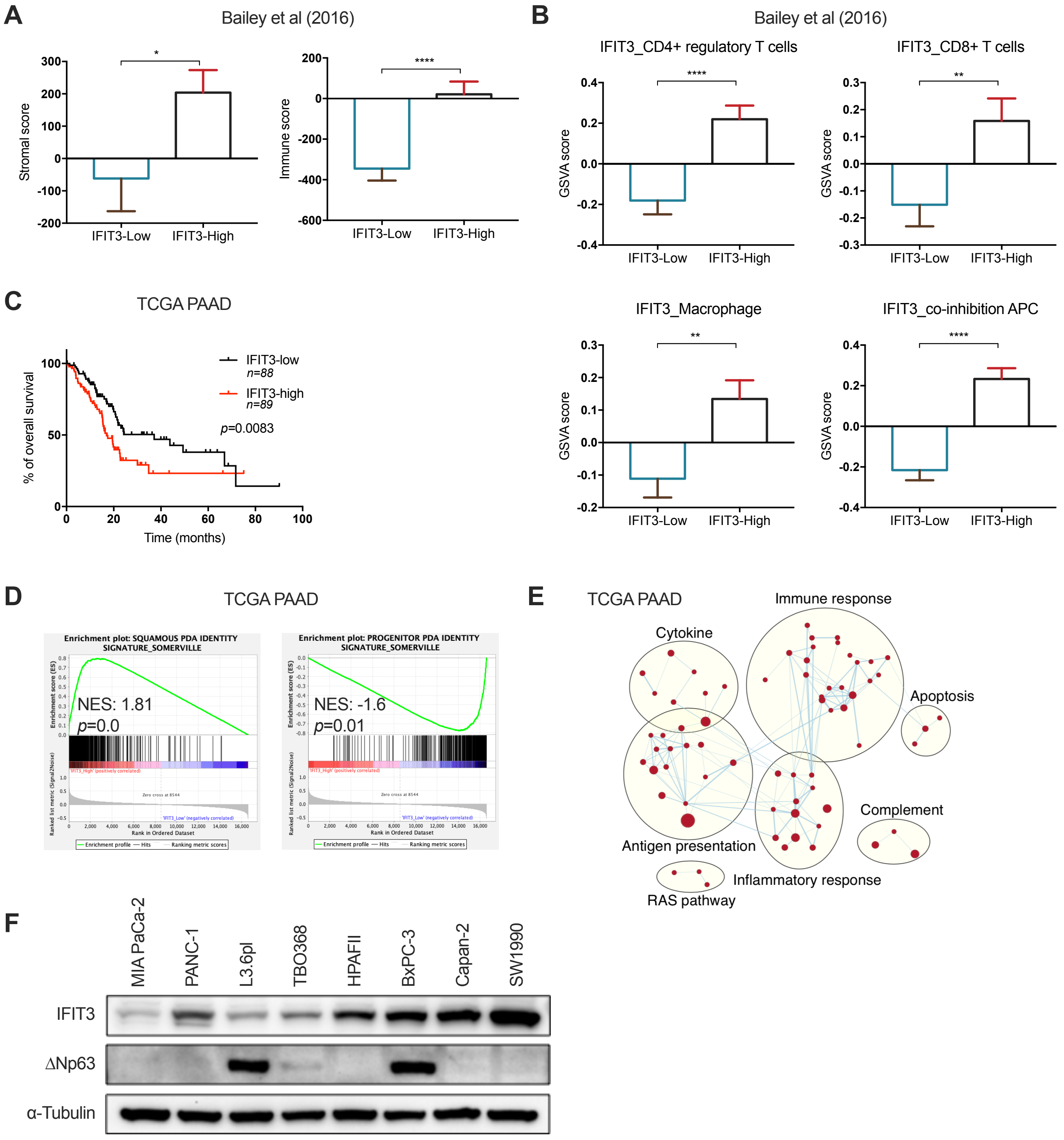

Figure S1 | Expression and characterization of IFIT3 in PDAC

Datasets from Bailey et al. and TCGA PAAD were downloaded and analyzed. Samples were stratified into quantiles based on the expression of IFIT3 (lower 50% and upper 50% of values). (A-B) IFIT3-High group is associated with higher stromal score and immune score. The sample GSVA score of different immune signatures were extracted from Bailey et al. (C) Kaplan–Meier survival analysis shows that IFIT3 expression is associated with poor survival of PDAC patients. (D) Gene set enrichment analysis shows enrichment of a squamous signature in the IFIT3-High group and progenitor signature in the IFIT3-Low group. (E) Enrichment map analysis shows that IFIT3 expression is characterized by the presence of inflammatory response, immune response, NF-κB pathway, cytokine and apoptosis. (F) The expression of IFIT3 and ΔNp63 were determined in a panel of PDAC cells. Membrane was stripped after IFIT3 detection and re-probed with α-Tubulin as loading control. *$p < 0.05$, **$p < 0.01$, ***$p < 0.001$, ****$p < 0.0001$.

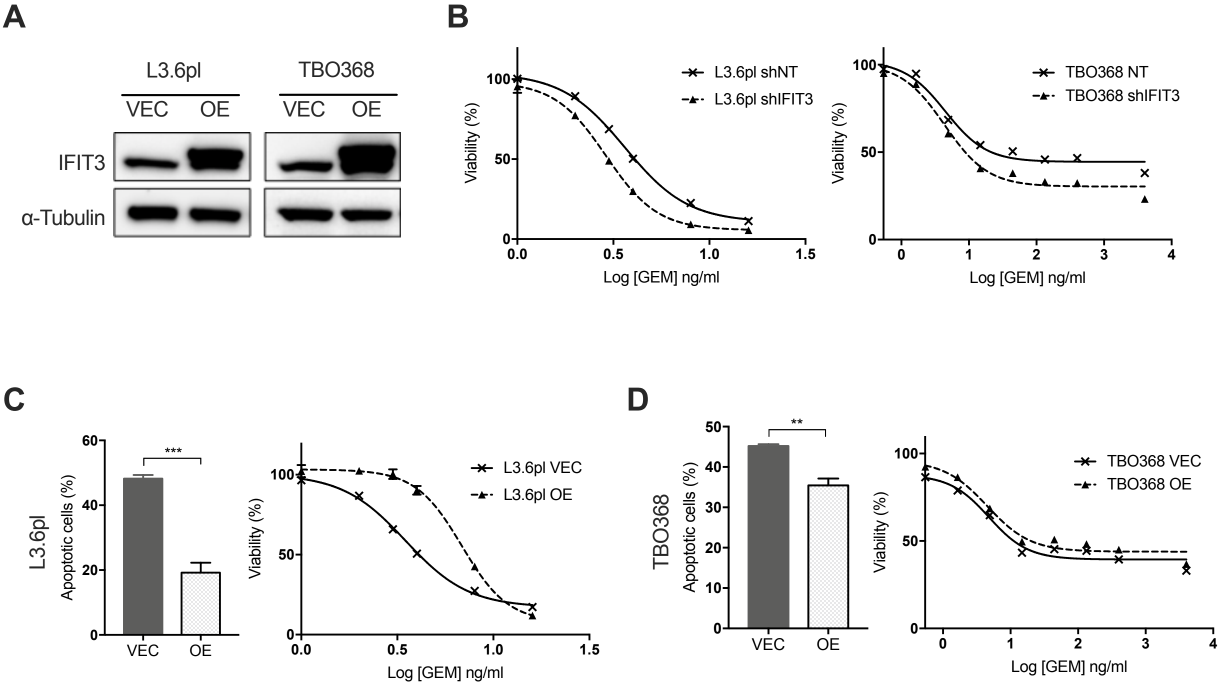

Figure S2 | IFIT3 renders chemotherapy resistance in PDAC cells

(A) IFIT3 overexpression was confirmed by western blot in both L3.6pl and TBO368 cells. Membrane was stripped after IFIT3 detection and re-probed with α-Tubulin. (B) IFIT3 knockdown sensitized PDAC cells to chemotherapy. MTT assay was used to determine cell viability after gemcitabine treatment for 48 h and 72 h, in L3.6pl and TBO368 respectively. (C-D) IFIT3 overexpression rendered chemotherapy resistance of PDAC cells. Apoptosis assay and MTT assay were used to determine apoptosis and cell viability after gemcitabine treatment for 48 h and 72 h, in L3.6pl and TBO368 respectively.

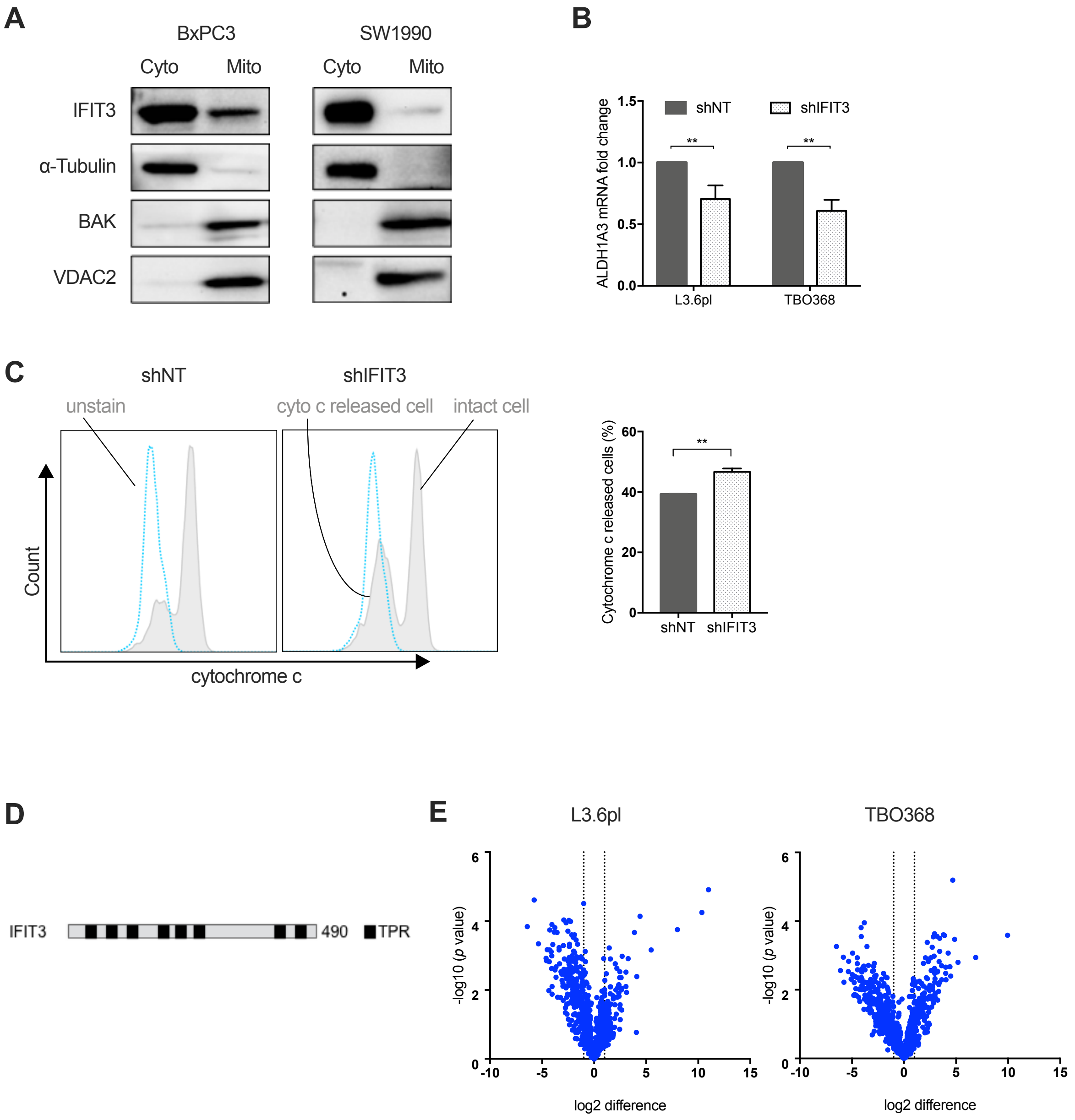

Figure S3 | IFIT3 regulates mitochondria associated apoptosis

(A) Western blot showed localization of IFIT3 in cytosol and mitochondria of BxPC3 and SW1990. Mitochondria were isolated as indicated in methods section. Cyto represent cytosol and Mito represent mitochondria. We probed α-Tubulin as cytosolic marker, BAK and VDAC2 as mitochondrial marker. (B) Knockdown of IFIT3 downregulated the expression of ALDH1A3 in L3.6pl and TBO368. (C) Quantitative cytochrome c release assay showed more cytochrome c released cells in IFIT3 knockdown cells compared to non-target control cells in L3.6pl. Cells were treated with gemcitabine for 48h before analysis. Representative histogram plots and bar graph are shown. (D) The structure of IFIT3 with multiple TPR motifs is illustrated. (E) Volcano plots illustrate statistically significant proteins binding to IFIT3 in L3.6pl and TBO368 treated with gemcitabine. Samples were immunoprecipitated with anti-IFIT3 antibody or normal IgG and sent for mass spectrometry analysis. Volcano plots display the –log10(*p* value) versus the log2 of the relative protein abundance to IgG control. Data are presented as mean ± SEM of three independent experiments. $^{**}p < 0.01$.

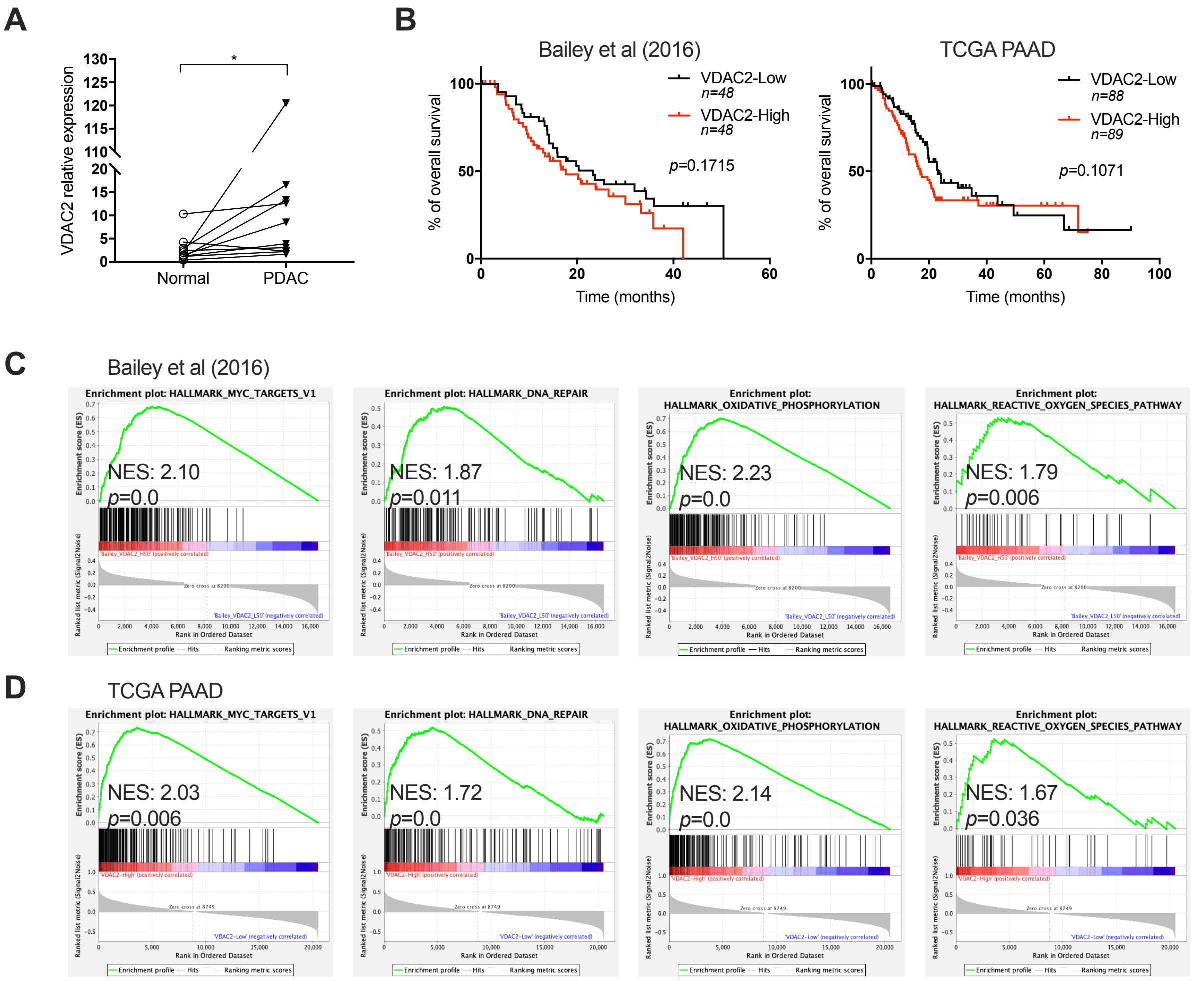

Figure S4 | Expression and characterization of VDAC2 in PDAC

(A) VDAC2 expression is higher in PDAC tissues as compared to adjacent normal tissues. Ten pairs of PDAC tissues and adjacent normal tissues were collected and analysed using qRT-PCR. 18s rRNA was used as internal control. (B-D) Dataset from Bailey et al. and TCGA PAAD were downloaded and analyzed. Samples were stratified into quantiles based on the expression of VDAC2 (lower 50% and upper 50% of values). (B) Kaplan-Meier survival analysis shows no significant difference between VDAC2-High and VDAC2-Low group in PDAC patients. Gene set enrichment analysis shows that VDAC2 expression is characterized by Myc targets, DNA repair, oxidative phosphorylation and oxygen species pathway in both Bailey's (C) and TCGA PAAD (D) datasets.

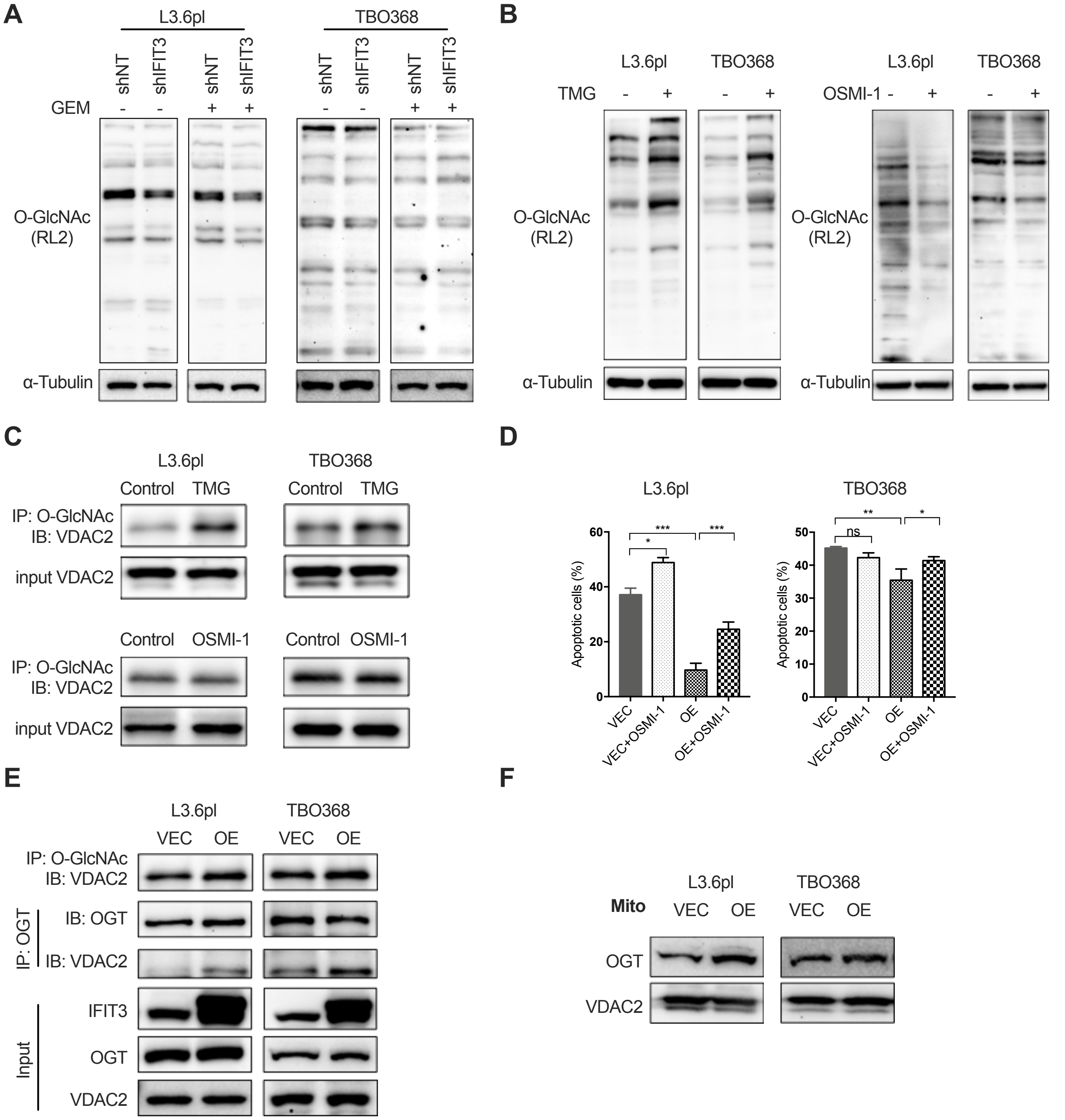

Figure S5 | IFIT3 modulates the O-GlcNAc level of VDAC2 through OGT

(A) Western blot demonstrated that knockdown of IFIT3 slightly decreased the total O-GlcNAc modification level in both L3.6pl and TBO368 cell lines, with or without gemcitabine treatment. Membrane was probed with O-GlcNAc (RL2), then stripped and re-probed with α-Tubulin. (B) TMG treatment increased the total O-GlcNAc level while OSMI-1 decreased O-GlcNAc level in L3.6pl and TBO368. Cells were treated for 24 h. TMG, 5 μM; OSMI-1, 20 μM. (C) TMG treatment increased the O-GlcNAc level of VDAC2 while OSMI-1 decreased O-GlcNAc level of VDAC2 in L3.6pl and TBO368. Cells were treated for 24 h. TMG, 5 μM; OSMI-1, 20 μM. (D) OSMI-1 increased gemcitabine induced apoptosis in L3.6pl and TBO368. Cells were treated as indicated for 48 h and 72 h, in L3.6pl and TBO368, respectively. OSMI-1, 20 μM. Data are presented as mean ± SEM of three independent experiments. (E) Immunoprecipitation of OGT showed more binding of VDAC2 after overexpression of IFIT3 in both L3.6pl and TBO368. (F) OGT level in mitochondrial fraction was increased in IFIT3 overexpression cells compared to control cells. Mitochondria were isolated as indicated in methods section. VDAC2 was used as loading control. *$p < 0.05$, **$p < 0.01$, ***$p < 0.001$, ns: non-significant, $p>0.05$.

Table S1 | Primers for qRT-PCR

Gene	Sequence (5’ to 3’)
GAPDH-for	GAAGGTGAAGGTCGGAGTC
GAPDH-rev	GAAGATGGTGATGGGATTTC
18s rRNA-for	GCTTAATTTGACTCAACACGGGA
18s rRNA-rev	AGCTATCAATCTGTCAATCCTGTC
IFIT1-for	GTGCTTGAAGTGGACCCTGA
IFIT1-rev	CCTGCCTTAGGGGAAGCAAA
IFIT2-for	CAATGCCTACCTGCATTGCC
IFIT2-rev	GAGCCACAGCGTGTCCTATT
IFIT3-for	AGTGAGGTCACCAAGAATTCCC
IFIT3-rev	GGCTGCCTCGTTGTTACCAT
NFKB1-for	AACAGAGAGGATTTCGTTTCCG
NFKB1-rev	TTTGACCTGAGGGTAAGACTTCT
NFKB3-for	GTGGGGACTACGACCTGAATG
NFKB3-rev	GGGGCACGATTGTCAAAGATG
IL6-for	CCTGAACCTTCCAAAGATGGC
IL6-rev	TTCACCAGGCAAGTCTCCTCA
XIAP-for	TATCAGACACCATATACCCGAGG
XIAP-rev	TGGGGTTAGGTGAGCATAGTC
RIG-I-for	TGCGAATCAGATCCCAGTGTA
RIG-I-rev	TGCCTGTAACTCTATACCCATGT
VDAC2-for	GCTACAGGACTGGGGACTTC
VDAC2-rev	AATGCCAAAACGAGTGCAGTT
OGT-for	CAGTAGCTTGGAGTAATCTTGGC
OGT-rev	GGTGACAGCCTTTTCAAAGTGAT
ALDH1A3-for	TGAATGGCACGAATCCAAGAG
ALDH1A3-rev	CACGTCGGGCTTATCTCCT
